# Diagnostic Challenges in Uncommon Firearm Injury Cases: A Multidisciplinary Approach

**DOI:** 10.3390/diagnostics15010031

**Published:** 2024-12-26

**Authors:** Andrea Vittorio Maria Failla, Gabriele Licciardello, Giuseppe Cocimano, Lucio Di Mauro, Mario Chisari, Francesco Sessa, Monica Salerno, Massimiliano Esposito

**Affiliations:** 1 Legal Medicine, Department of Medical, Surgical and Advanced Technologies, “G.F. Ingrassia”, University of Catania, 95123 Catania, Italy; avmfailla@gmail.com (A.V.M.F.); licciardello.gabriele@gmail.com (G.L.); dr.luciodimauro@gmail.com (L.D.M.); monica.salerno@unict.it (M.S.); 2 Department of Mental and Physical Health and Preventive Medicine, University of Campania “Vanvitelli”, 80121 Napoli, Italy; giuseppe.cocimano@unicampania.it; 3 “Rodolico-San Marco” Hospital, Santa Sofia Street, 87, 95121 Catania, Italy; m.chisari@policlinico.unict.it; 4 Faculty of Medicine and Surgery, “Kore” University of Enna, 94100 Enna, Italy; massimiliano.esposito@unikore.it

**Keywords:** firearm injuries, forensic pathology, multidisciplinary approach, radiological diagnostics, ballistics, forensic autopsy

## Abstract

**Background**: Firearm wounds tend to have a precise pattern. Despite this, real-world case presentations can present uncertain elements, sometimes deviating from what is considered standard, and present uncommon features that are difficult for forensic pathologists and ballistic experts to explain. **Methods**: A retrospective analysis of autopsy reports from the Institute of Legal Medicine, University of Catania, covering 2019–2023, included 348 judicial inspections and 378 autopsies performed as part of the institute’s overall activities. Among these, seventeen cases of firearm deaths were identified, with three atypical cases selected for detailed analysis. An interdisciplinary approach involving forensic pathology, radiology, and ballistics was used. **Results**: The selected cases included: (1) A 56-year-old female with a thoracic gunshot wound involving three 7.65 caliber bullets, displaying complex trajectories and retained bullets; (2) A 48-year-old male with two cranial gunshot injuries, where initial evaluation suggested homicide staged as a suicide, later confirmed to be a single self-inflicted shot; and (3) A 51-year-old male was found in a car with two gunshot wounds to the head, involving complex forensic evaluation to distinguish between entrance and exit wounds and determine trajectory. The findings showed significant deviations from standard patterns, underscoring the critical role of radiological imaging and ballistic analysis in understanding wound morphology and projectile trajectories. **Conclusions**: This case series highlights the necessity for standardized yet adaptable protocols and cooperation among forensic specialists. A flexible approach allows forensic investigations to be tailored to the specific circumstances of each case, ensuring that essential examinations are conducted while unnecessary procedures are avoided. Comprehensive data collection from autopsies, gross organ examinations, and, when needed, radiological and histological analysis is essential to accurately diagnose injuries, trace bullet trajectories, retrieve retained projectiles, and determine the fatal wound, particularly in complex cases or those involving multiple shooters.

## 1. Introduction

Firearm injuries pose a significant challenge in forensic investigations due to their complex and often unpredictable nature. Traditional forensic literature provides detailed descriptions of wound morphology, expected injury patterns, and associated physical evidence, guiding forensic pathologists in their investigations [1,2,3]. However, real-world cases frequently deviate from these models, presenting unique challenges that necessitate a comprehensive and multidisciplinary approach. The intersection of forensic pathology, radiology, ballistics, and genetics offers a robust framework for examining these unusual cases [4,5,6].

Autopsies and radiological imaging (X-ray, CT, MRI) allow detailed analysis of wounds, tissue damage, and bullet trajectories, precisely locating fragments and determining projectile paths [7,8,9,10,11,12]. Ballistics complements these analyses by identifying the firearm and ammunition, reconstructing the incident, and understanding impact dynamics [13,14]. Although DNA analysis on fired cartridges and casings is challenging due to heat, pressure, and metal ion degradation, recent techniques have improved efficiency in obtaining genetic profiles [15,16,17,18].

Atypical firearm injuries pose unique forensic challenges: irregular wound morphology may result from bullet deformation, intermediary targets, or ricochet effects, altering trajectories and producing misleading features; close-range shootings can introduce backspatter contamination, while internal deflections through bones or secondary trauma complicate injury analysis; heat damage from high-velocity bullets and mixed mechanisms of injury further obscure ballistic interpretation, underscoring the need for a multidisciplinary approach [1,2,18,19].

Through a retrospective analysis, this study focused on a case series aiming to analyze the complexities of unusual firearm injuries, emphasizing the importance of a multidisciplinary forensic approach. By examining three atypical cases from the Institute of Legal Medicine at the University of Catania, Italy, the study highlights the challenges of such injuries and underscores the need for standardized protocols to enhance the accuracy and reliability of forensic investigations.

## 2. Materials and Methods

### 2.1. Inclusion and Exclusion Criteria

In this study, the selection criteria for unusual firearm cases were as follows: a single cartridge used in the firearm incident, presence of an unusual firearm injury, and the execution of external examination, radiological imaging, autopsy, and ballistic investigation. Cases were excluded if they were incomplete, involved broken ammunition, or lacked an unusual firearm injury.

The study was conducted in accordance with the Declaration of Helsinki. Since the procedures performed were part of the standard work-up for such cases and given the medicolegal context where these procedures are conducted by legal authorities, approval from next-of-kin was not required. Furthermore, the privacy and personal identity information of all participants were protected, and all data were analyzed anonymously.

### 2.2. Case Selection

This study is based on a retrospective analysis of all autopsy reports from the Institute of Legal Medicine at the University of Catania, Italy, covering a five-year period from 2019 to 2023. The dataset includes a total of 348 judicial inspections and 378 judicial autopsies. The total number of judicial inspections and forensic autopsies were compared to exclude duplicates, identifying a total of 580 unique cases handled between 2019 and 2023. After this initial screening, 17 cases of fatal firearm injuries were identified. Based on the inclusion and exclusion criteria, only three cases with significant unusual lesions were included in the present case series.

## 3. Results

Table 1 summarizes the main characteristics of the 17 selected cases of fatal firearm injuries.

### 3.1. Case 1: Thoracic Gunshot Wound with Retained Bullet

**Figure 1:**
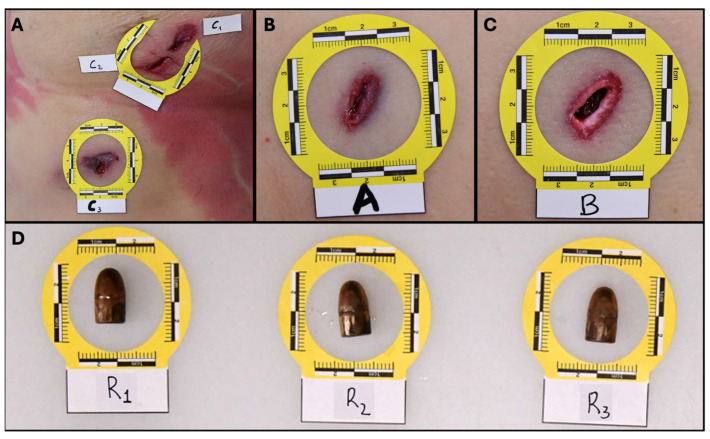
**Thoracic gunshot wounds and retained bullets relative to Case 1.** (**A**) Three distinct wounds from Bullet C are documented: Entrance Wound C1 (lower left breast), Exit Wound C2 (adjacent to C1), and Entrance Wound C3 (lower chest). The wounds show varied characteristics, with C1 and C3 presenting inwardly folded edges and reddish-brown discoloration, while C2 displays outwardly folded edges, indicating it as an exit wound; (**B**) Entrance Wound A from Bullet A located on the left back at the level of the first lumbar vertebra. The wound is oval-shaped, with finely hemorrhagic edges and surrounded by a reddish-brown area and an ecchymotic halo; (**C**) Entrance Wound B from Bullet B, positioned at the left back at the level of the seventh thoracic vertebra, shows a triangular shape with inwardly folded edges, surrounded by a reddish-brown area and an ecchymotic halo.; (**D**) Retained bullets (R1, R2, R3) corresponding to each shot fired. These retained bullets were confirmed through CT scans and autopsy findings, supporting the conclusion that all wounds, except C2, represent entrance wounds. The irregular wound shapes suggest instability and rotation of the bullets during flight, leading to side-on impacts.

The first case involves a 56-year-old female who sustained three gunshots to the chest. The shooter fired three 7.65 caliber bullets from approximately 3–8 m. The shots were fired from different positions: one from the front and two from behind. Below is a detailed description of the wounds, ordered cranio-caudally (Figure 1).

In Figure 1A, three distinct wounds from Bullet C are documented. Entrance Wound C1 is located on the lower left breast. This wound is oval-shaped, measuring approximately 0.8 cm by 0.5 cm, with inwardly folded, finely hemorrhagic edges. The surrounding skin presents a reddish-brown discoloration extending about 2.5 cm by 1.2 cm. Exit Wound C2, positioned near C1, is also irregularly oval (1.8 cm by 0.5 cm), with outwardly folded edges. It appears continuous with C1 and is surrounded by a similar reddish-brown area. Entrance Wound C3 penetrates the chest and presents as a round wound approximately 0.6 cm in diameter. This wound exposes soft tissue embedded with a metallic foreign body. The surrounding area displays a reddish-brown discoloration (2.2 cm by 1.4 cm) accompanied by a bluish discoloration (5.5 cm by 2.5 cm).

In Figure 1B, Entrance Wound A from Bullet A is noted on the left back at the level of the first lumbar vertebra. The wound is oval-shaped, measuring 1.2 cm by 0.5 cm, with inwardly folded, finely hemorrhagic edges. Surrounding it is a reddish-brown area approximately 1 cm in diameter, encircled by a 3 mm ecchymotic halo.

In Figure 1C, Entrance Wound B from Bullet B is located on the left back at the level of the seventh thoracic vertebra. This wound is triangular, with a base measuring 0.5 cm and a height of 1.3 cm. The edges are inwardly folded, and a reddish-brown area (1.5 cm) and a 2 mm ecchymotic halo surround the wound.

CT scans and autopsy findings, as shown in Figure 1D, confirmed that all wounds, except C2, represent entrance wounds, and all bullets were retained within the body. The wound shapes suggest that the bullets likely lost stability and rotated during flight, resulting in side-on impacts against the skin rather than typical tip-first impacts.

### 3.2. Case 2: Complex Cranial Gunshot

**Figure 2:**
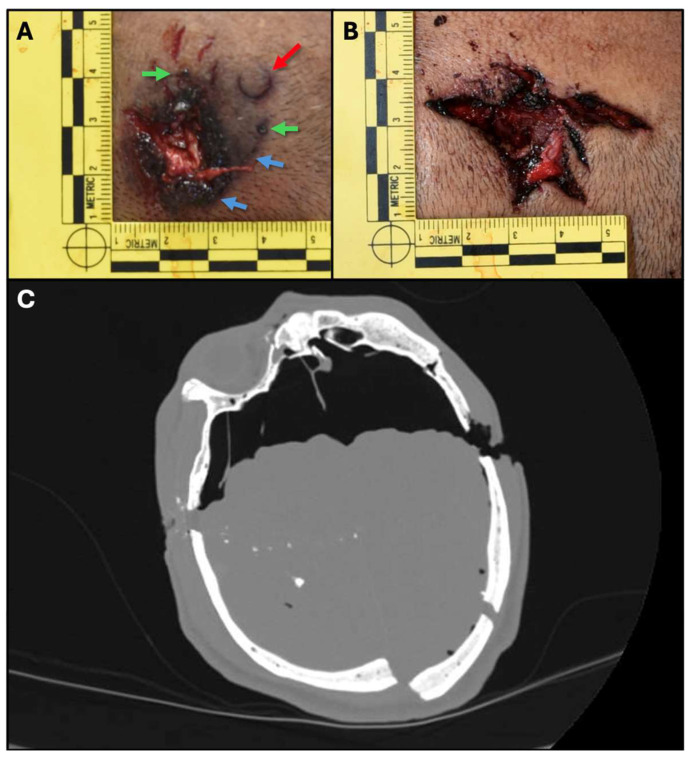
**Cranial Gunshot Wounds and CT Scan of Case 2.** (**A**) Entrance wound caused by Bullet A, located on the right temporal region of the head. The wound is irregularly oval with stellate margins, featuring three small notches, and measures approximately 1.6 cm by 1.2 cm. The wound exposes brain tissue, with a visible protruding flap of dura mater and a surrounding dark brownish discoloration measuring 3.2 cm by 3.5 cm. Key forensic indicators are present, highlighted by colored arrows: Werkgartner’s sign is indicated by a red arrow, showing the imprint of the pistol’s recoil spring guide rod; a burn halo and soot deposition are marked by blue arrows; and unburned powder residues are identified with green arrows around the wound; (**B**) Exit wound caused by Bullet B on the left occipital region. This wound is characterized by hemorrhagic, thickened, and irregular stellate margins with six distinct arms, measuring approximately 5.3 cm by 3.5 cm, exposing underlying skull and brain tissue; (**C**) CT scan showing intracranial bone fragments in a “comet tail” pattern, characteristic of an entrance wound. The scan provided crucial information to differentiate between entrance and exit wounds, supporting the conclusion that Wound A was the entrance wound while Wound B was the exit wound.

In the second case, we examine a 48-year-old right-handed male who was found deceased at a crime scene with a single gunshot to the head. A wound was located at the right temple, and a second wound was at the left occipital region. A 9 × 19 caliber firearm was found near the body, which was lying partially on its right side, causing the right wound to be covered in blood (Figure 2).

In Figure 2A, Bullet A inflicted an entrance wound located on the right temporal region. The wound is irregularly oval with slightly stellate margins, featuring three small notches measuring 0.5 cm, 0.3 cm, and 0.3 cm. The dimensions of the wound are approximately 1.6 cm by 1.2 cm, exposing brain tissue and showing an outwardly protruding flap of dura mater. A dark brownish discoloration measuring 3.2 cm by 3.5 cm surrounds this wound.

Figure 2B displays Bullet B’s exit wound on the left temporal region. This wound has hemorrhagic, thickened, and irregular stellate margins with six distinct arms measuring around 5.3 cm by 3.5 cm. The wound exposes the skull and brain tissue beneath.

Identifying the entrance wound was essential, given the initial suspicion that this case may have involved a homicide staged as a suicide, particularly as the victim was right-handed, and the right temporal wound was initially obscured by blood. CT scans, along with a thorough macroscopic examination, confirmed that the initially suspected entrance wound (Wound B) was, in fact, the exit wound. This conclusion was supported by several forensic indicators associated with Wound A:

- Werkgartner’s sign was visible under Wound A, showing the imprint of the pistol’s recoil spring guide rod.
- A burn halo was observed around Wound A.
- Soot deposition was present around the wound.
- Two unburned powder residues were identified in the skin surrounding Wound A.
- A comet tail pattern of intracranial bone fragments, characteristic of entrance wounds, originated from Wound A.

The thickened margins of Wound B were attributed to the evaporation of water content from the skin due to prolonged exposure to air and sunlight, as illustrated in Figure 2B. This combination of forensic findings allowed for a clear differentiation between the entrance and exit wounds, critical for understanding the circumstances surrounding the death.

### 3.3. Case 3: Gunshot Wounds in a Vehicle

In this case, a right-handed individual was found deceased inside a car in a public place, with two gunshot wounds to the head. One wound was located in the upper front neck area, and the other in the frontal region of the skull. The body was seated in the passenger seat with a firearm in the right hand, resting in the lap, and the head facing forward. The neck wound was partially obscured by blood.

**Figure 3:**
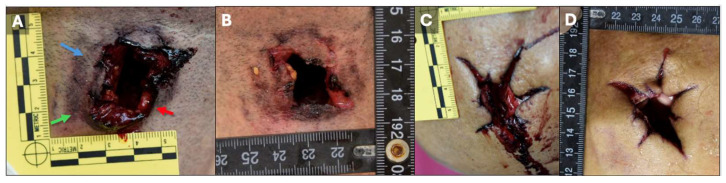
**Gunshot Wounds Observed in Case 3.** (**A**) Entrance wound caused by Bullet A, located on the upper front of the neck, near the midline and approximately 2 cm from the chin. The wound is irregular in shape, measuring about 2.5 cm by 2 cm, with hemorrhagic, diastatic (separated) margins. Surrounding the wound are several forensic indicators: blue arrow indicates a tattoo halo, green arrow points to soot deposition, and red arrow shows a burn halo; (**B**) Entrance wound caused by Bullet A, showing additional detail from a different angle, with clearly visible hemorrhagic and separated margins; (**C**) Exit wound caused by Bullet B, located in the frontal region of the skull, near the midline. The wound features hemorrhagic, thickened, and irregular stellate margins with five distinct arms, with the largest extending approximately 2.5 cm; (**D**) Another view of the exit wound caused by Bullet B, showing the stellate margins and the surrounding hemorrhagic area. The overall dimensions of the wound are approximately 5.5 cm by 4.5 cm, with exposed skull and brain tissue.

In Figure 3A,B, Bullet A created an entrance wound located on the upper front of the neck, near the midline and about 2 cm from the chin. The wound was irregular in shape, measuring approximately 2.5 cm by 2 cm, with hemorrhagic, separated (diastatic) margins, and was surrounded by a reddish-brown area extending about 3 cm by 2.5 cm.

Figure 3C,D show Bullet B’s exit wound in the frontal region of the skull, close to the midline. This wound had hemorrhagic, thickened, and irregularly stellate margins with five distinct arms, the largest extending approximately 2.5 cm. The overall wound dimensions were around 5.5 cm by 4.5 cm, with exposed skull and brain tissue visible.

**Figure 4:**
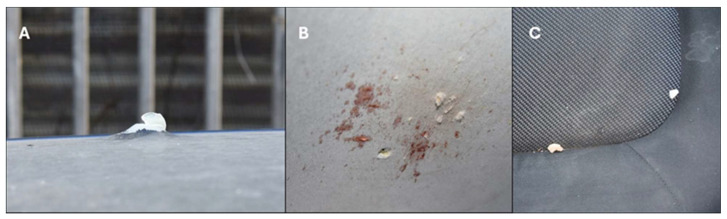
(**A**) hole with outward-oriented edges in the car roof; (**B**) hole in the car roof with bloodstain area; (**C**) cranial bone fragments found on the car seat.

Due to the positioning of the body and the nature of the wounds, distinguishing between a potential suicide and a staged homicide was essential, as illustrated in Figure 4.

A thorough examination, along with evidence from the judicial inspection, confirmed that Wound A was consistent with an entrance wound. Several critical factors led to this determination:

- The discovery of a hole in the car roof, with outward-oriented metal edges.
- Bone fragments matching the cranial vault found on the back seats of the vehicle.
- Bloodstains are present on the inner roof surface near the hole.
- Presence of a burn halo, soot deposition, and tattooing around Wound A.

These observations, along with the forensic examination, helped establish the origin and nature of the wounds, clarifying the circumstances of the death.

## 4. Discussion

Firearm injuries often display common morphological features that aid in their identification and analysis. These include entrance and exit wounds, tissue damage, and ballistic trauma patterns that are extensively documented in forensic literature [1,2,20,21]. However, real-world cases sometimes deviate from these models, presenting atypical characteristics that complicate forensic analysis. The inherent variability in the presentation of firearm injuries can lead to significant challenges. Factors such as the type of firearm, ammunition used, distance of the shot, and intermediary objects can influence the wound patterns, making it difficult to apply standard forensic principles [22,23]. The case series presented in this study offers unique insights into the complexities of firearm injuries, often deviating from standard descriptions found in forensic literature. This discussion compares our findings with prior research, highlighting the need for a multidisciplinary approach.

Forensic pathology plays a crucial role in understanding the cause and manner of death in firearm injury cases. Forensic pathologists conduct a meticulous external examination of the cadavers and forensic autopsies, where they examine wound morphology, tissue damage, and physical evidence to elucidate injury mechanisms. This detailed examination includes the analysis of entry and exit wounds, the assessment of wound tracks through the body, and the evaluation of internal organ damage [7,8,9]. The integration of histological studies allows for the microscopic examination of tissue samples, providing insights into the type and extent of injuries sustained. In particular, histological and immunohistochemical investigations are considered fundamental both for determining the time since death and for assessing the vitality of the lesion, thereby excluding the possibility that the wound was inflicted postmortem [24,25]. This comprehensive approach helps in identifying bullet trajectories, determining the sequence of injuries, and, in cases involving multiple shooters, ascertaining which shot was fatal. By combining macroscopic and microscopic findings, forensic pathologists contribute essential information that aids in the legal investigation and reconstruction of shooting dynamics.

Traditional forensic pathology texts describe predictable entry wounds as small, circular defects with abrasion collars and exit wounds as larger and more irregular due to the bullet’s deformation and pressure release. Tissue damage typically includes laceration, crushing along the bullet track, and cavitation effects from the projectile’s kinetic energy. In Case 3, the presence of soot and stippling around Wound A confirmed a close-range shot, consistent with traditional descriptions. In Case 1, the bullet wounds displayed significant deviations from these models. The cranio-caudal arrangement of wounds C1, C2, and C3 indicated instability of the bullets, leading to irregular impacts. This aligns with Haag et al., who noted that bullets that ricochet or are destabilized by impact with an object can produce atypical entry wounds due to their yawing or tumbling motion in flight [26].

Radiology, particularly forensic radiology, is essential in the investigation of firearm injuries [10,11,12]. Radiological imaging techniques such as X-rays, CT scans, and MRI provide a non-invasive means to visualize internal injuries, locate bullet fragments, and assess the trajectory of projectiles within the body [27,28]. These imaging modalities, in addition to autopsy findings, offer a detailed view of the skeletal and soft tissue damage caused by gunshots [29,30]. CT scans, for example, are highly effective in identifying the exact location of lodged bullets and assessing their potential movement within the body. The ability to reconstruct 3D images from radiological data aids in visualizing the bullet’s path, which is critical for understanding the dynamics of the shooting and matching it with the observed injuries. Additionally, radiological imaging can reveal subtle fractures, tissue disruptions, and other internal damage that might not be immediately visible during the autopsy, thus enhancing the overall forensic analysis [31]. Our use of CT scans in Case 2 highlighted the detailed skeletal and soft tissue damage, which is consistent with findings by Thali et al. [20]. Jeffery et al. further demonstrated the effectiveness of CT scans in identifying the exact location of lodged bullets and assessing potential movement [32]. In Case 3, postmortem CT confirmed the trajectories and helped distinguish between entrance and exit wounds. Ampanozi et al. highlighted the importance of postmortem CT in identifying occult gunshot wounds and understanding their trajectory [33].

Ballistic experts provide crucial insights into the type of firearm and ammunition used in shooting incidents [13,14]. They examine bullet trajectories, determine the angle and distance of shots, and reconstruct the sequence of events leading to the injuries. This involves examining the physical characteristics of bullets and cartridge cases, studying gunshot residue patterns, and using advanced techniques such as ballistic gel testing and high-speed photography to simulate and understand the behavior of projectiles upon impact [34,35]. The expertise of ballistic analysts is vital in establishing the connection between the firearm and the injuries sustained, which can help in identifying the shooter and corroborating witness testimonies [34]. Their analyses can distinguish between entrance and exit wounds, understand the effects of different types of ammunition, and provide a scientific basis for courtroom testimony. The comprehensive study of ballistics not only supports the forensic pathologist’s findings but also enhances the overall investigation by providing a clear picture of the shooting dynamics [13,14]. The unusual trajectories in Cases 1 and 3 emphasized the need for detailed ballistic evaluations. Kneubuehl et al. focused on wound ballistics, highlighting how projectiles can behave unpredictably within the body, especially when encountering bone [36]. Stefanopoulos et al. discuss the impact of projectile-tissue interaction, including bullet tumbling, deformation, and fragmentation, on wound characteristics [37]. In Case 2, the identification of bullet types and their impact on wound morphology underscored the necessity of a multidisciplinary approach. Shrestha’s work underscores the importance of identifying bullet types and their impact on wound morphology, which necessitates a multidisciplinary approach [38]. The forensic evaluation of gunshot wounds involves understanding the physical and chemical effects of projectiles, the documentation of wound characteristics, and the differentiation between entry and exit wounds. Forensic pathologists collaborate with ballistics experts, law enforcement, and medical professionals to accurately determine the type of firearm used, the distance and direction of fire, and the overall impact on the victim’s body [38].

The necessity for a multidisciplinary approach is evident from our case studies. Forensic pathology alone could not provide a comprehensive understanding of the injury mechanisms observed. Radiology and ballistics provided critical insights that were essential for an accurate reconstruction of the incidents. This approach is supported by literature advocating interdisciplinary collaboration to enhance the accuracy and reliability of forensic investigations [39].

**Figure 5:**
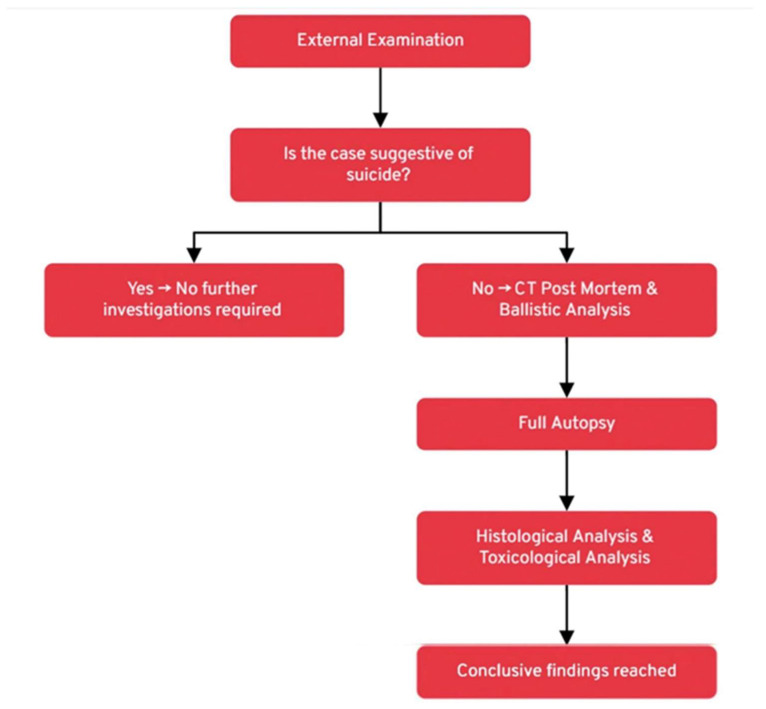
Flowchart summarizing the structured forensic investigation process for firearm-related deaths.

The complexity of firearm injuries requires a structured and sequential approach to forensic investigation, as seen in the presented cases. This approach combines parallel and consecutive examinations to ensure a comprehensive analysis (Figure 5).

Initially, an external examination is conducted to assess whether the case is suggestive of suicide. If the findings support this hypothesis, further investigations may not be necessary. However, in cases where the dynamics are unclear or a homicidal scenario is suspected, a postmortem CT scan is performed in parallel with a ballistics analysis. These steps provide essential data on bullet trajectories and the dynamics of the shooting, which guide the subsequent full autopsy. The autopsy then provides a macroscopic evaluation of the injuries, while histological and toxicological analyses are conducted in parallel to further clarify the microscopic characteristics of the wounds and identify any substances that may have influenced the death. Through this methodical process, forensic investigators are able to gather all relevant data necessary to reconstruct the events surrounding the death and reach conclusive findings, thus ensuring that no further investigations are required.

Looking at future prospects, the impact of new technologies will facilitate greater integration among these fields, providing improved tools for forensic investigations. AI technologies have the potential to significantly advance diagnostic capabilities in forensic pathology, radiology, and ballistics. AI systems can be trained to detect and classify injuries, such as gunshot wounds, more accurately and rapidly than traditional methods [40,41,42,43,44]. These systems can identify subtle patterns in imaging data that might be missed by human examiners, leading to improved diagnostic accuracy.

AI can improve diagnostic accuracy even from the analysis of photographic documentation collected during external cadaveric examinations: recently, Cheng et al. explored the feasibility of using a deep learning model to classify entry and exit gunshot wounds in digital color images [45]. Digging deeper into evaluating the impact of AI in other fields, pathologists are already integrating AI into the recognition of histological slides, reducing the workload and excluding a large percentage of non-cancerous slides [46].

Convolutional neural networks (CNNs) are a form of deep learning used for image classification across various fields, including radiology and pathology. Garland et al. explored the application of CNNs for classifying gross postmortem images of visceral organs, demonstrating that CNNs could classify postmortem images with over 95% accuracy [47]. This integration shows great potential for advancements in forensic pathology and could be applied to firearm lesions in the future. AI-powered tools could soon be able to analyze histological slides to identify tissue damage and pathological changes caused by gunshot wounds, providing a detailed assessment of injuries.

In the field of forensic radiology, particularly through machine learning and deep learning algorithms, AI can significantly enhance the interpretation of radiological images. Recent studies have demonstrated the potential of AI in this domain. For instance, Garland et al. explored the application of CNNs to differentiate fatal head injuries from non-head injuries using postmortem CT images, achieving promising accuracy rates (70–92.5%) [48]. Additionally, Yasaka & Abe discussed the high performance of CNNs in recognizing various conditions in radiological images, suggesting their application could greatly assist radiologists in achieving diagnostic excellence [49]. Furthermore, deep learning techniques have been applied to detect and analyze traumatic injuries in postmortem imaging, enhancing the accuracy and efficiency of forensic examinations [50]. These advancements highlight the significant potential of AI in improving forensic radiology practices. AI could automate the detection and segmentation of bullet fragments and bone fractures in CT and MRI scans.

In the field of ballistics, AI can enhance the matching process of ballistics evidence by analyzing unique marks left on bullets and cartridge cases. Machine learning models can compare these marks against large databases to identify the firearm used in a crime [51]. The study by Kudonu et al. examines the application of AI in firearm examination to provide an objective analysis of bullets and cartridge cases, moving beyond traditional subjective methods. Using a pre-trained CNN, the researchers aimed to evaluate the potential of AI in analyzing class characteristics such as rifling marks and individual characteristics of bullets [52].

AI can facilitate the integration of diverse data sources, such as autopsy reports, radiological images, and ballistics evidence, into a cohesive case management system. This integrated approach can improve the efficiency and accuracy of forensic investigations. AI-driven platforms can compile and analyze data from multiple forensic disciplines, providing investigators with comprehensive insights and aiding in the identification of patterns and correlations.

## 5. Conclusions

The three cases presented in this study highlight the complexities and challenges of atypical firearm injuries in forensic investigations. These cases deviated from standard wound patterns, emphasizing the limitations of relying solely on traditional forensic methodologies. Our findings underscore the critical importance of an interdisciplinary approach, integrating forensic pathology, radiology, and ballistics to accurately interpret unusual wound characteristics and reconstruct the events leading to death. Employing a collaborative framework allows forensic experts to effectively analyze complex cases, ensuring thorough examinations and precise determinations of cause and manner of death. This approach enhances the reliability of forensic conclusions and supports the legal process by providing comprehensive and accurate evidence. The study advocates for the adoption of adaptable protocols tailored to the specific circumstances of each case. Flexibility in applying forensic tools and techniques enables professionals to utilize appropriate resources without unnecessary procedures, improving efficiency and outcomes. In conclusion, embracing a multidisciplinary and flexible approach is essential for addressing the evolving complexities of firearm injuries in forensic practice. This strategy not only improves the accuracy and reliability of forensic findings but also contributes to the advancement of forensic science and the effective administration of justice.

## Figures and Tables

**Table 1:** The main characteristics (sex, age, dynamic, the presence of entrance/exit wound, the weapon) for each case were reported.

Case n.	Sex Age	Dynamic	Entrance Wound	Exit Wound	Weapon
1	Female (F) 56 years old (y.o.)	Homicide	Chest, left. Atypical Back, right. Atypical Back, right. Atypical	None	Semi-automatic pistol(cal. 7.65)
2	Male (M) 48 y.o.	Suicide	Cranial, right temporal bone. Atypical	Cranial, left parietal bone. Atypical	Semi-automatic pistol (cal. 9 × 19)
3	M. 51 y.o.	Suicide	Neck, upper front. Atypical	Cranial, frontal bone. Atypical	Semi-automatic pistol (cal. 9 × 19)
4	F. 48 y.o.	Homicide	Cranial, right temporal bone. Typical Cranial, right zygomatic bone. Typical	None	Revolver (cal. 45 ACP)
5	F. 49 y.o.	Homicide	Cranial, occipital bone. Typical	Cranial, orbital region. Typical	Revolver (cal. 45 ACP)
6	M. 63 y.o.	Suicide	Cranial, right temporal bone. Typical	Cranial, left zygomatic bone. Typical	Revolver (cal. 45 ACP)
7	M. 45 y.o.	Homicide	Chest, left. Typical	Chest, left. Typical	Semi-automatic pistol (cal. 9 × 21)
8	M. 26 y.o.	Homicide	Cranial, left temporal bone. Typical	None	Semi-automatic pistol (cal. 38 SPL)
9	M. 47 y.o.	Homicide	Cranial, frontal bone. Typical Cranial, occipital bone. Typical	Cranial, parietal bone. Typical	Unknown
10	M. 33 y.o.	Suicide	Cranial, frontal bone. Typical	None	Revolver (cal. 38 SPL)
11	F. 44 y.o.	Suicide	Cranial, right temporal bone. Typical	Cranial, left parietal bone. Typical	Revolver (cal. 38 SPL)
12	M. 46 y.o.	Homicide	Cranial, parietal bones, midline. Typical Chest, left. Typical Right shoulder. Typical	None	Semi-automatic pistol(cal. 7.65)
13	M. 30 y.o.	Suicide	Cranial, right parietal bone. Typical	Cranial, left parietal bone. Typical	Semi-automatic pistol(cal. 9 × 21)
14	F. 27 y.o.	Homicide	Cranial, left parietal bone. Typical Back, right. Typical	Cranial, nose. Typical Chest, right. Typical	Semi-automatic pistol(cal. 9 × 21)
15	M. 90	Suicide	Cranial, right temporal bone. Typical	Cranial, left parietal bone. Typical	Semi-automatic pistol(cal. 6.35 Browning)
16	M. 86 y.o.	Suicide	Cranial, right temporal bone. Typical	Cranial, left parietal bone. Typical	Semi-automatic pistol (cal. 9 × 21)
17	M. 68 y.o.	Suicide	Cranial, right temporal bone. Typical	Cranial, left parietal bone. Typical	Semi-automatic pistol(cal. 7.65)

## Data Availability

The original contributions presented in this study are included in the article. Further inquiries can be directed to the corresponding author.
